# Age-Related Olfactory Decline Is Associated With Levels of Exercise and Non-exercise Physical Activities

**DOI:** 10.3389/fnagi.2021.695115

**Published:** 2021-07-26

**Authors:** Giorgia Sollai, Roberto Crnjar

**Affiliations:** Department of Biomedical Sciences, Section of Physiology, University of Cagliari, Cagliari, Italy

**Keywords:** elderly adults, physical activities, lifestyle, olfactory function, odor discrimination and identification

## Abstract

**Objective**: This cross-sectional study evaluates the impact of active or non-active lifestyle in terms of physical, cognitive and social activity on the olfactory function in Elderly Subjects (ES) and aims at looking for a correlation between the time devoted to life activities and the score obtained during the olfactory tests by each individual.

**Methods**: One hundred and twenty-two elderly volunteers were recruited in Sardinia (Italy) and divided into active ES (*n* = 60; 17 men, 43 women; age 67.8 ± 1.12 years) and inactive ES (*n* = 62; 21 men, 41 women, age 71.1 ± 1.14 years) based on their daily physical activities. The olfactory function was evaluated using the “*Sniffin’s Sticks*” battery test, while the assessment of daily activities was made by means of personal interviews.

**Results**: A significant effect of active or inactive lifestyle was found on the olfactory function of ES (*F*_(1,120)_ > 10.16; *p* < 0.005). A positive correlation was found between the olfactory scores and the number of hours per week dedicated to physical activities (Pearson’s *r* > 0.32, *p* ≤ 0.014) in both active and inactive ES.

**Conclusions**: High levels of exercise and non-exercise physical activity are strongly associated with the olfactory function and, consequently, with the quality of life of the elderly. Given the limited physical exercise of elderly people, they can benefit from a more active lifestyle by increasing non-exercise physical activities.

## Introduction

The sense of smell plays an important role in the identification of environmental dangers (smoke, noxious gas, chemicals, spoiled or burnt foods), in social relationships, in eating behavior, and in food choices (Stevenson, 2010; Schubert et al., 2013; Attems et al., 2015). Humans show a great inter-individual variability in their olfactory perception, both of complex odors and single molecules, due to environmental and genetic factors (Keller et al., 2007; Menashe et al., 2007; Calderón-Garcidueñas et al., 2010; Sorokowska et al., 2013; Sollai et al., 2019, 2020; Melis et al., 2021). In addition to chronic diseases such as neurodegenerative, inflammatory/immune, cardiovascular, metabolic, depressive, renal, nasal, and hepatic ones (Graves et al., 1999; Larsson et al., 1999; Seiberling and Conley, 2004; Boesveldt et al., 2008; Ross et al., 2008; Doty, 2009; Wilson et al., 2009; Steinbach et al., 2011; Palouzier-Paulignan et al., 2012; Perricone et al., 2013; Attems et al., 2014; Croy et al., 2014b; Doty and Kamath, 2014; Makizako et al., 2014; Stuck and Hummel, 2015; Sollai et al., 2021), one of the factors that mainly affects the olfactory function is the natural aging process (Doty et al., 1984; Cain and Stevens, 1989; Min et al., 2021) and the progressive sensory deterioration with age (Schubert et al., 2017). Population-based studies of olfactory loss indicate that the maximum olfactory performance occurs between the 3rd and the 5th decade and that a reduced olfactory function is very common in elderly populations, affecting more than 50% of individuals aged between 65 and 80 years and 62–80% of the elderly over 80 years (Doty et al., 1984; Attems et al., 2015). The causes are not well known, but previous studies highlighted a relationship between the difficulties of Elderly Subjects (ES) to identify odors and a decrease of their general cognitive abilities, such as aging-linked deficits in memorizing odors, a faster decline in perceptual speed, and episodic memory (Larsson et al., 2000; Hedner et al., 2010; Dintica et al., 2019).

Olfactory dysfunction significantly affects nutritional status, quality of life, physical well-being, daily safety as well as mortality (Croy et al., 2014a; Pinto et al., 2014; Attems et al., 2015). People suffering from olfactory disorders, at all ages from young to elderly, report a negative impact on their mental and emotional health, increased social isolation, inability to protect themselves from environmental dangers, dissatisfaction with eating, and problems in feeding behavior (Hummel and Nordin, 2005; Boesveldt and Parma, 2021; Min et al., 2021).

Physical activity is known to directly and positively affect many risk factors for cardiovascular and neurodegenerative diseases, diabetes, adiposity and obesity, some types of cancer, some aspects of mental health, poor quality of life, and mortality (Blair et al., 1996; Shephard and Balady, 1999; Tuomilehto et al., 2001; Franco et al., 2005; Friedenreich et al., 2010; Umpierre et al., 2011; Wen et al., 2011; Buchman et al., 2012; Das and Horton, 2012; Hallal et al., 2012; Strasser, 2013). Individuals may also benefit from a modest activity: in fact, compared to inactive individuals, those who were even weakly active (about 1.5 h per week) lived 3 years longer (Wen et al., 2011). The benefits of exercise are particularly evident in older populations where, in addition to reducing the risk of cardiovascular disease and improving physical fitness (Franco et al., 2005; Lang et al., 2007), regular exercise relieves depression, protects against neurodegeneration and dementia, improves learning, memory, and executive function, counteracting age-related mental decline and protecting brain areas crucial for higher cognitive processes from atrophy (Colcombe and Kramer, 2003; Heyn et al., 2004; Weuve et al., 2004; Podewils et al., 2005; Cotman et al., 2007). In general, exercise has been associated with maintaining good physical and mental health in the old age and increasing lifespan (Burke et al., 2001; Franco et al., 2005; Middleton et al., 2008; Schubert et al., 2013). Previous studies have also emphasized a relationship between the ability to identify odors and the social life of individuals (Boesveldt et al., 2017). In particular, it has been found that body odor can convey the age, health, and emotional state of individuals (de Groot et al., 2012; Mitro et al., 2012; Olsson et al., 2014). Furthermore, most social interactions involve the act of eating and drinking, the pleasure of which can be seriously impaired and limited by a reduced olfactory function, leading individuals to limit their social interactions (Murphy, 2008; Boesveldt et al., 2017). Finally, several studies have sought and highlighted an association between cognitive decline and olfactory impairment, especially in odor identification which requires certain cognitive functions such as memory and the use of vocabulary (Wilson et al., 2007; Schubert et al., 2008; Dintica et al., 2019; Suzuki et al., 2021).

Most of the studies on the relationship between olfactory function and physical activity and/or lifestyle present a longitudinal design that evaluates olfactory function before, during, and after a period of years in the same ES. Presently, it is not clear whether the health benefits deriving from exercise also extend to a lower incidence of olfactory impairment, commonly associated with age (Schubert et al., 2013). Therefore, given the high percentage of elderly people with a reduced olfactory function (Murphy et al., 2002; Bramerson et al., 2004; Vennemann et al., 2008; Schubert et al., 2012), the low awareness of the impact that a decline in olfaction may have on safety, nutrition, and quality of life in the elderly (Nordin et al., 1995; Miwa et al., 2001; Santos et al., 2004; Hummel and Nordin, 2005), it is important to identify the modifiable factors associated with the relationship between olfactory function and aging.

On this basis, in the current study, our objective was to determine the impact of lifestyle on olfactory function in elderly and disease-free subjects that could affect their olfactory perception. Thus, by means of a cross-sectional study, we compared the abilities to perceive, discriminate and identify odors in a population of active ES in terms of physical, cognitive, and social activity, with those of ES adapted to a little or no active lifestyle. Furthermore, we looked for a correlation between the time devoted to life activities and the score obtained during the olfactory tests by each individual in both populations.

## Materials and Methods

### Subjects

The elderly volunteers (*n* = 122) who took part in this study were recruited in the metropolitan area of Cagliari and in the province of South Sardinia (Italy) and were divided into two groups. The first group consisted of active ES (*n* = 60; 17 men, 43 women; age 67.8 ± 1.12 years) who reported having an active and stimulating lifestyle, in terms of physical, social, and cognitive activity; the second group, on the other hand, was made up of elderly adults who reported leading a less active life both from a physical, social and cognitive point of view (*n* = 62; 21 men, 41 women, age 71.1 ± 1.14 years), and were therefore classified as inactive ES. The two groups were matched by age (χ^2^ = 0.17, *p* = 0.68) and gender (χ^2^ = 0.44, *p* = 0.51). The inclusion criteria were: healthy subjects aged >55 years and belonging to both sexes, with a self-esteemed normal olfactory function, or in any case who did not report having problems with their perception of odors. The exclusion criteria were: past or current diagnosis of cancer, neurodegenerative diseases, schizophrenia, depression, autoimmune/inflammatory diseases, severe cardiovascular disease, diabetes, head trauma, acute/chronic rhinosinusitis, nasal surgery, respiratory distress, and difficulty to understand the aim of the study and the protocol used. Comprehensive information on lifestyle, environmental and behavioral factors such as smoking history, exposure to cigarette smoke at home or at work, use of alcohol, physical, social and cognitive activity (intended as hours dedicated to reading, writing, or solving puzzle games) were self-reported and obtained from all subjects through questionnaires. Significant differences between the two ES groups in relation to this comprehensive information are shown in Supplementary Table 1.

### Assessment of Physical, Social, and Cognitive Activity

Assessment of physical, social, and cognitive activities was made by means of personal interviews. In particular, the subjects were asked: (a) “how many hours a day do you spend walking for exercise or running, strenuous housework or gardening, working in the fields, swimming or dancing?” (b) “How many hours a day do you dedicate to social activities such as meetings, organizing and/or participating in events, attending seminars?”; (c) “how many hours a day do you spend reading a book and/or solving puzzles?”; and (d) “for each of these activities, how many days a week?.” The hours spent in each of these activities were summed and expressed as hours of activity per week (hrs/week). Furthermore, according to Buchman et al. (2012), physical activity (intended as motor activity) is defined as exercise physical activity, while the hours dedicated to social and cognitive activities are considered together and defined as non-exercise physical activities.

### Olfactory Sensitivity Assessment

The orthonasal olfactory function of each subject was evaluated by means of the “*Sniffin’s Sticks*” battery test (Hummel et al., 1997), consisting of three subtests for olfactory threshold (*T*-test), odor discrimination (*D*-test), and odor identification (*I*-test). For the *T*-test, the experimenter has at his/her disposal a kit of 48 pens grouped in 16 triplets, each consisting of two pens containing a solvent and a third pen (target pen) filled with a solution of n-butanol at increasing concentrations. If the subject identifies the target pen twice in succession, a reversal of the scale begins. The test ends when the 7th scale reversal is completed and the *T*-test score is given by the average of the last four reversals. For *D*-test, the experimenter has 16 triplets available, each consisting of two pens containing the same odor and one filled with a different odor (target pen). The participant’s goal is to identify the target pen. The *D*-test score corresponds to the number of correct answers from 0 to 16. For *I*-test, individuals have to smell 16 pens containing familiar odors. Each time the participant smells the odor contained in a pen, he/she has to choose one out of four different items in a forced-choice procedure. The score corresponds to the number of correct identifications from 0 to 16.

The total TDI olfactory score is obtained from the sum of the scores from the *T*-test, *D*-test, and *I*-test. The normalized values for age and gender reported in Hummel et al. (2007), were used to classify each subject as normosmic or hyposmic both by taking into account the total TDI score and that obtained with each of the three sub-tests (*T*-test, *D*-test, *I*-test).

### Statistical Analysis

Fisher’s exact test was used to determine the presence of a significant difference in the distribution of subjects classified as normosmic or hyposmic based on the olfactory TDI score and those of the *T*-test, *D*-test, and *I*-test, between the two populations of ES taken into consideration.

One-way ANOVA was used to check for a significant difference between the two populations (active ES vs. inactive ES) in the total TDI olfactory score and in the T, D, and I scores. *Post hoc* tests were performed with the Fisher’s LSD test; *p*-values <0.05 were considered significant. The statistical analysis was carried out using STATISTICA for WINDOWS (version 7.0; StatSoft Inc, Tulsa, OK, USA).

The Pearson’s correlation coefficient was used to evaluate the association between two variables of interest: TDI, T, D, or I olfactory score vs. hrs/week of exercise or non-exercise physical activities, for each population, separately. Statistical analyses were performed using GraphPad Prism 6 (GraphPad Software, San Diego, CA, USA). *P*-values < 0.05 were considered to be significant.

## Results

**Figure 1:**
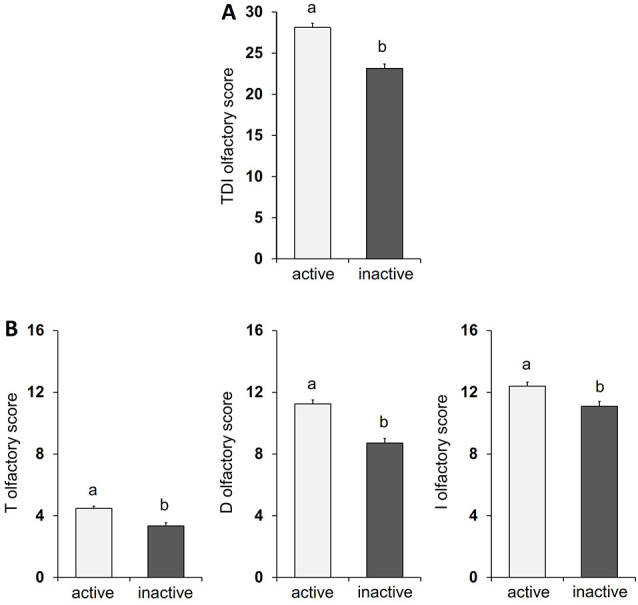
Mean value ± SEM of the TDI olfactory score **(A)** and of the olfactory threshold (T), odor discrimination (D), and odor identification (I) scores **(B)** obtained by active HS (*n* = 60) and inactive HS (*n* = 62). Different letters indicate significant differences between lifestyles (*p* < 0.05; Fisher’s LSD test subsequent to one-way ANOVA).

The mean ± SE value of the total TDI olfactory score and of that obtained with each of the specific sub-tests for olfactory threshold (T), odor discrimination (D), and odor identification (I), both from active ES and inactive ES, are shown in Figure 1. In detail, one-way ANOVA revealed a significant effect of active vs. inactive lifestyle on all the olfactory scores considered (TDI score: *F*_(1,120)_ = 47.09, *p* < 0.0001, effect size 1.000; T score: *F*_(1,120)_ = 20.43, *p* < 0.0001, effect size 0.994; D score: *F*_(1,120)_ = 41.86, *p* < 0.0001, effect size 1.000; I score: *F*_(1,120)_ = 10.16, *p* = 0.0018, effect size 0.885) and *post hoc* comparisons indicated that the scores obtained by active ES is significantly higher than that obtained by inactive ES (*p* < 0.005).

Table 1 shows the distribution of both active and inactive ES classified as normosmic or hyposmic according to their TDI, T, D, and I olfactory status. Fisher’s method showed that the percentage of active ES classified as normosmic or hyposmic is significantly different compared to inactive ES (χ^2^ > 6.53; *p* ≤ 0.011). In particular, we found that about 72% of active ES and only 25% of inactive ES were normosmic. Moreover, just 10% of active ES were hyposmic for T, D, and I olfactory status as compared to 25–44% of inactive ES.

**Table 1:** Distribution of the active and inactive Elderly Subjects (ES) classified as normosmic or hyposmic based on their overall olfactory status (TDI), Threshold (T), Discrimination (D), or Identification (I) status.

	Group	ES active	ES inactive	*p*-Value
Variable	Olfactory status	*n* (%)	*n* (%)	
TDI	Normosmic	43 (71.67)	16 (25.81)	<0.000
	Hyposmic	17 (28.33)	46 (74.19)	
T	Normosmic	53 (88.33)	39 (62.90)	<0.001
	Hyposmic	7 (11.67)	23 (37.10)	
D	Normosmic	55 (91.67)	35 (56.45)	<0.000
	Hyposmic	5 (8.33)	27 (43.55)	
I	Normosmic	55 (91.67)	46 (74.19)	<0.011
	Hyposmic	5 (8.33)	16 (25.81)	

*p-Value derived from Fisher’s Exact Test. Active ES subjects (*n* = 60) and inactive ES subjects (*n* = 62)*.

**Figure 2:**
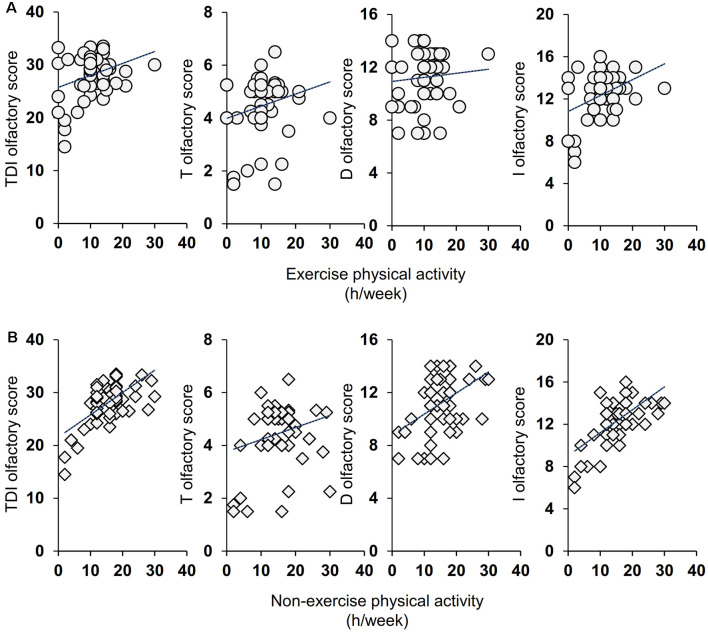
Correlation analysis between the TDI, T, D, and I olfactory score and the number of hours per week (h/week) dedicated by each active elderly subjects (ES) to the exercise **(A)** and non-exercise **(B)** physical activities.

**Figure 3:**
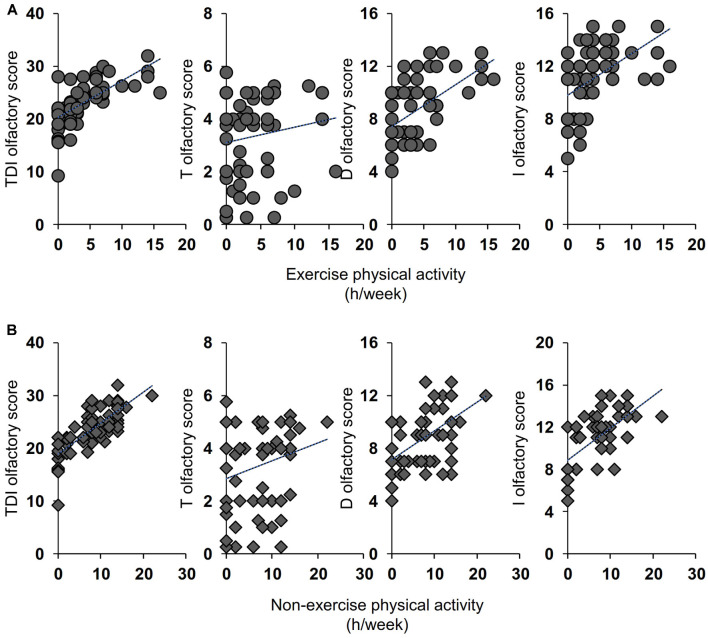
Correlation analysis between the TDI, T, D, and I olfactory score and the number of hours per week (h/week) dedicated by each inactive ES to the exercise **(A)** and non-exercise **(B)** physical activities.

Pearson’s correlation test was used to ascertain whether the number of hours per week dedicated to life activities (exercise and non-exercise) was correlated with the olfactory scores obtained by individuals. As shown in Figure 2, for the active ES the results indicate that a positive correlation exists both between the TDI and I olfactory scores obtained by each individual with his/her exercise physical activity (Pearson’s *r* = 0.32, *p* = 0.014 and Pearson’s *r* = 0.40, *p* = 0.002 for TDI and I score, respectively) and with his/her non-exercise physical activity (Pearson’s *r* = 0.64, *p* < 0.0001 and Pearson’s *r* = 0.63, *p* < 0.0001 for TDI and I score, respectively). A positive correlation was also found between D olfactory score and non-exercise physical activity (Pearson’s *r* = 0.46, *p* = 0.0002), while the statistical analysis did not show any correlation between T olfactory score and life physical activities, both for exercise (Pearson’s *r* = 0.22, *p* = 0.10) and non-exercise (Pearson’s *r* = 0.24, *p* = 0.07). Figure 3 shows the results obtained with the Pearson’s correlation test for the inactive ES. In particular, we found a positive correlation between both exercise and non-exercise physical activities and TDI olfactory score (Pearson’s *r* = 0.69, *p* < 0.0001 and Pearson’s *r* = 0.78, *p* < 0.0001, for exercise and non-exercise activities, respectively), D score (Exercise activities: Pearson’s *r* = 0.57, *p* < 0.0001; Non-exercise activities: Pearson’s *r* = 0.53, *p* < 0.0001) and I score (Exercise activities: Pearson’s *r* = 0.52, *p* < 0.0001; Non-exercise activities: Pearson’s *r* = 0.66, *p* < 0.0001). On the other hand, no correlation was found between both life activities and the olfactory threshold score (Exercise activities: Pearson’s *r* = 0.16, *p* = 0.21; Non-exercise activities: Pearson’s *r* = 0.24, *p* = 0.06).

## Discussion

Physical and cognitive activities are known to improve health conditions by slowing down the physiological aging of all body systems. In particular, a minor decline has been observed in the functionality of systems such as the cardiovascular, muscular, skeletal, respiratory, and nervous ones (Morris et al., 1953; Blair et al., 1996; Shephard and Balady, 1999; Umpierre et al., 2011; Wen et al., 2011; Das and Horton, 2012). As regards the nervous system, several studies have documented that the olfactory acuity decreases with age and that the elderly, for this reason, often manifest bad eating habits that can lead to malnutrition, weight gain, depression, social isolation, and total or partial inability to detect environmental hazards (Duffy et al., 1995; Temmel et al., 2002; Croy et al., 2014a; Boesveldt and Parma, 2021).

Based on these considerations, the main objective of this study with a cross-sectional design was to verify whether an active lifestyle, both from a physical and a cognitive point of view, is related to the olfactory performance of elderly adults. The results indicate that older individuals who were classified as physically and mentally little active or totally inactive reach lower TDI, T, D, and I olfactory scores than older adults who were classified as active, and a higher number of subjects was classified as hyposmic for all olfactory performances. In fact, the percentage of inactive ES classified as hyposmic was between 26% (for I olfactory status) and 74% (for TDI olfactory status), while that of active ES was between 8% (for D and I olfactory status) and 28% (for TDI olfactory status). These results suggest a double interpretation. On the one hand, inactive subjects seem to show an olfactory dysfunction precisely because they are resigned to a lifestyle that is poor in terms of exercise and non-exercise activities; on the other hand, they show little interest in the environment around them because they are not stimulated by the odors that characterize it. It is reasonable to assume that olfactory impairment may lead elderly individuals to reduce their social relationships due to their inability to enjoy the pleasures of food (e.g., in a situation of conviviality with other people such as a dinner with friends, a party with relatives, et cetera) and/or due to insecurity linked to their own body odor (Temmel et al., 2002; Boesveldt et al., 2017; Boesveldt and Parma, 2021). In fact, previous studies have shown that people with reduced olfactory abilities experience less pleasure in food and eating and that a variable percentage (19–41%) of patients with olfactory dysfunction report not being able to perceive the odor of their own body, to have problems with their personal hygiene and with the use of perfumes (Miwa et al., 2001; Temmel et al., 2002; Blomqvist et al., 2004; Nordin et al., 2011). This reduced participation in social life can make people more prone to depression (Croy et al., 2014b). Given the fact that several studies have documented a relationship between lifestyle and depression, individuals who are not very active or totally inactive may tend to develop depressive attitudes that can affect the areas of the brain responsible for cognitive abilities that are also involved in the higher olfactory function.

The second objective of this study was to verify the presence of a positive relationship between the olfactory scores reached by each elderly subject of both groups and the weekly hours dedicated to exercise and non-exercise physical activities. The positive relationship that we found between olfactory performance and exercise/non-exercise physical activities may have two interpretations. First, staying active slows down the normal aging process including that of the central nervous system. In fact, exercise has been reported to increase synaptic plasticity, by acting on the synaptic structure and enhancing synaptic efficiency, as well as strengthening neurogenesis, metabolism, and vascular function (Cotman et al., 2007). These aspects have been well documented in the hippocampus, the area of the limbic system where the olfactory memory is located. So, in active individuals, synaptic plasticity improves and this keeps their brains younger, by counteracting cognitive decay and depressive aspects that affect the same areas where the olfactory memory is located (Cotman et al., 2007; Schubert et al., 2013; Attems et al., 2015). We found that the differences between active and inactive ES are related to the ability to discriminate and identify odors that require the involvement of higher brain functions (Hedner et al., 2010), according to the fact that the ability to identify odors requires a successful recalling of information from memory and suggesting that inactive ES seem to be unable to do it. Although memory may show a general weakening in the elderly, it is strongly affected by episodic olfactory presentation (Cain and Stevens, 1989). This aspect is particularly evident from the correlation results we have obtained. In both groups, we found a positive correlation between TDI, D, and I olfactory scores with both exercise and non-exercise physical activities, but not with the olfactory threshold score, thus suggesting that life activities may be associated with the loss of cognitive functions of the brain areas. In agreement, not only exercise activities but also higher levels of non-exercise activity were found to be associated with cognitive abilities in aged adults (Buchman et al., 2012). Second, exercise reduces the risk factors for certain diseases such as diabetes, hypertension, metabolic disorders, depression, obesity, and inflammatory diseases, which converge to cause brain dysfunction and neurodegeneration (Franco et al., 2005; Cotman et al., 2007; Buchman et al., 2012; Hamer et al., 2012) and are pathologies associated with a reduction in the olfactory function (Graves et al., 1999; Ross et al., 2008; Wilson et al., 2009; Perricone et al., 2013; Croy et al., 2014a; Pinto et al., 2014; Kim et al., 2019; Sollai et al., 2021); this may explain why among active older adults we observed both a higher number of normosmic subjects and higher olfactory scores.

This study has several strengths. First, our results show a clear association between physical activities (both exercise and non-exercise) and normosmic or hyposmic olfactory status in the elderly. In particular, physical, cognitive, and social activities are associated with a better performance in odor discrimination and odor identification, which are reflected in a better overall olfactory function in the elderly who practice these activities. Second, our results show a strong correlation between the number of hours dedicated to daily activities and the olfactory scores obtained by the participants, especially those categorized as weakly active or inactive. However, this study has some limitations. First, the investigations relating to the physical and cognitive status of the elderly, which lack evaluations obtained with standardized tests, were performed exclusively through questionnaires. Second, this is a cross-sectional study that evaluates the instantaneous olfactory function; this means that an evaluation of the olfactory function in relation to the lifestyle of the elderly over time is precluded. Future studies will be necessary to investigate these relationships prospectively in order to confirm the associations we have observed.

In conclusion, the results of this study show a strong relationship between exercise and non-exercise physical activity and olfactory function, with a better olfactory performance by those elderly who have an active lifestyle. Given the association between olfactory function and social relationships, eating habits, attention to dangers, and cognitive decline, these results suggest that a good sense of smell can improve the quality of life of elderly people. These findings have important implications not only for observational studies but also for the design of those aimed at intervening on physical activity and cognitive abilities in the old age. Older individuals, for whom physical exercise proper may be limited due to other health problems, can still benefit from a more active lifestyle by increasing non-exercise physical activities.

## Data Availability Statement

The raw data supporting the conclusions of this article will be made available by the authors, without undue reservation.

## Ethics Statement

The studies involving human participants were reviewed and approved by Ethical Committee of the University Hospital of Cagliari. The patients/participants provided their written informed consent to participate in this study.

## Author Contributions

GS conducted the experiment, wrote the manuscript, and analyzed the data. GS and RC conceived the experiment. RC revised the manuscript. All authors contributed to the article and approved the submitted version.

## Conflict of Interest

The authors declare that the research was conducted in the absence of any commercial or financial relationships that could be construed as a potential conflict of interest.

## Publisher’s Note

All claims expressed in this article are solely those of the authors and do not necessarily represent those of their affiliated organizations, or those of the publisher, the editors and the reviewers. Any product that may be evaluated in this article, or claim that may be made by its manufacturer, is not guaranteed or endorsed by the publisher.
