# Supplementary material for: Age-Related Olfactory Decline Is Associated With Levels of Exercise and Non-exercise Physical Activities
Source: Front Aging Neurosci. 2021 Jul 26;13:695115. doi: 10.3389/fnagi.2021.695115 (PMC8423134; doi:10.3389/fnagi.2021.695115)
Supplement: Supplementary file 1 [file Data_Sheet_1.PDF]

Table S1. Distribution of the active and inactive Elderly Subjects (ES) in relation to smoking history, exposure to cigarette smoke at home or at work, use of alcohol, physical, social and cognitive activity.

Variable	Group	Active ES	Inactive ES	χ^2	p-value
Smoker	Yes	2	5	1.26	0.26
	No	58	57		
Exposure to cigarette smoke	Yes	14	13	0.10	0.75
	No	46	49		
Use of alcohol	Yes	11	10	0.10	0.75
	No	49	52		
Physical activity	Yes	52	28	23.27	<0.0001
	No	8	34		
Social activity	Yes	26	10	10.85	0.001
	No	34	52		
Cognitive activity	Yes	57	42	14.81	0.0001
	No	3	20		

p-Value derived from Fisher's Exact Test. Active ES (n = 60) and inactive ES (n = 62). Significant differences are highlighted in red.

Table S2. Pearson's correlation coefficient results on the association between TDI, T, D or I olfactory score *vs*. hrs/week of exercise or non-exercise physical activities, for each population, separately. Significant correlation are highlighted in red.

Active Elderly Subjects				
**Life activities**	**Olfactory score**	**r**	**R^2**	**p**
Exercise	TDI	0.32	0.10	0.014
	T	0.22	0.05	0.10
	D	0.08	0.01	0.54
	I	0.40	0.16	0.002
Non-exercise	TDI	0.64	0.41	< 0.0001
	T	0.24	0.06	0.07
	D	0.46	0.21	0.002
	I	0.63	0.40	< 0.0001

Inactive Elderly Subjects				
**Life activities**	**Olfactory score**	**r**	**R^2**	**p**
Exercise	TDI	0.69	0.47	< 0.0001
	T	0.16	0.03	0.21
	D	0.57	0.33	< 0.0001
	I	0.52	0.27	< 0.0001
Non-exercise	TDI	0.78	0.60	< 0.0001
	T	0.24	0.06	0.06
	D	0.53	0.28	< 0.0001
	I	0.66	0.44	< 0.0001
